# Temporal trends in anticoagulation use and clinical outcomes among medicare beneficiaries with non-valvular atrial fibrillation

**DOI:** 10.1007/s11239-023-02838-2

**Published:** 2023-08-02

**Authors:** Brett D. Atwater, Jennifer D. Guo, Allison Keshishian, Rachel Delinger, Cristina Russ, Lisa Rosenblatt, Jenny Jiang, Huseyin Yuce, Mauricio Ferri

**Affiliations:** 1 Inova Heart and Vascular Institute, 4Th Floor Medical Directors Suite, 3300 Gallows Road, Falls Church, VA 22042 USA; 2 Bristol-Myers Squibb Company, Lawrenceville, NJ USA; 3 STATinMED, LLC, Ann Arbor, MI USA; 4 Pfizer Inc., New York, NY USA; 5 New York City College of Technology, City University of New York, New York, NY USA

**Keywords:** Atrial fibrillation, Direct oral anticoagulant, Major bleeding, Stroke/systemic embolism

## Abstract

**Purpose:**

Oral anticoagulants effectively prevent stroke/systemic embolism among patients with non-valvular atrial fibrillation but remain under-prescribed. This study evaluated temporal trends in oral anticoagulant use, the incidence of stroke/systemic embolism and major bleeding, and economic outcomes among elderly patients with non-valvular atrial fibrillation and CHA_2_DS_2_–VASc scores ≥ 2.

**Methods:**

Retrospective analyses were conducted on Medicare claims data from January 1, 2012 through December 31, 2017. Non-valvular atrial fibrillation patients aged ≥ 65 years with CHA_2_DS_2_–VASc scores ≥ 2 were stratified by calendar year (2013–2016) of care to create calendar-year cohorts. Patient characteristics were evaluated across all cohorts during the baseline period (12 months before diagnosis). Treatment patterns and clinical and economic outcomes were evaluated during the follow-up period (from diagnosis through 12 months).

**Results:**

Baseline patient characteristics remained generally similar between 2013 and 2016. Although lack of oral anticoagulant prescriptions among eligible patients remained relatively high, utilization did increase progressively (53–58%). Among treated patients, there was a progressive decrease in warfarin use (79–52%) and a progressive increase in overall direct oral anticoagulant use (21–48%). There were progressive decreases in the incidence of stroke/systemic embolism 1.9–1.4 events per 100 person years) and major bleeding (4.6–3.3 events per 100 person years) as well as all-cause costs between 2013 and 2016.

**Conclusions:**

The proportions of patients with non-valvular atrial fibrillation who were not prescribed an oral anticoagulant decreased but remained high. We observed an increase in direct oral anticoagulant use that coincided with decreased incidence of clinical outcomes as well as decreasing total healthcare costs.

**Supplementary Information:**

The online version contains supplementary material available at 10.1007/s11239-023-02838-2.

## Highlights

- Between 2013 and 2016, there was rapid and progressive temporal trends toward DOAC uptake supplanting warfarin prescriptions
- This trend coincided with progressive reductions in stroke/SE, MB, and healthcare costs.
- However, the proportions of overall OAC use increased by less than 10%, possibly indicating persistent OAC underutilization.
- Further study is needed to determine where gaps in treatment may remain.

## Introduction

Atrial fibrillation (AF) is the most common cardiac arrhythmia in the United States, and the risk of developing AF increases with age [1]. AF incidence, prevalence, and AF-attributable mortality have increased in the US as the population has aged [1–3]. AF is estimated to currently affect as many as 6 million Americans and is expected to affect 12 million by 2030 [4, 5]. People with AF are at a five-fold greater risk of stroke than the general population [6]. Stroke contributes to the excess morbidity, mortality, and healthcare costs observed among the AF patient population—particularly those aged ≥ 65 years [7].

Historically, the oral anticoagulant (OAC) warfarin has been the standard of care for stroke prevention among patients with non-valvular AF (NVAF). But warfarin is associated with increased risk of bleeding and requires dietary restrictions and consistent laboratory monitoring, all of which have resulted in the underutilization of warfarin and contributed to excess stroke among patients with NVAF [8, 9]. Since 2010, the United States Food & Drug Administration has approved four direct OACs (DOACs) developed to address this treatment gap: dabigatran (2010), rivaroxaban (2011), apixaban (2012), and edoxaban (2015) [10–13]. Based on clinical trial evidence of noninferior or superior stroke prevention with generally lower bleeding rates among DOACs vs warfarin, 2014 clinical guidelines recommended DOACs over warfarin for patients with NVAF and risk factors for stroke, with the recommendation reiterated in 2019 [14, 15]. Since their introduction into clinical practice, DOACs have been associated with overall similar or lower risk of stroke and generally lower risk of major bleeding (MB) as compared with warfarin [16, 17]. After the inclusion of DOACs in clinical guidelines, their utilization has increased, while warfarin use has decreased [18].

Nonetheless, underutilization of OAC treatment in clinical practice has been reported from the period before the introduction of DOACs through early post-market surveillance [18–22]. Moreover, this underutilization has been associated with adverse outcomes [23, 24]. The timing of these prior studies in relation to DOAC rollouts underscores the importance of understanding the evolving impact of DOACs on real-world NVAF treatment patterns and associated outcomes. Several real-world studies have observed shifts toward guideline-recommended DOAC treatment for NVAF in Europe, Australia, and East Asia^.^[25–33]. Some included observations of associated reductions in stroke and MB incidence [27, 30, 33]. Some US studies have observed temporal trends in OAC use around the time of new guidelines and DOAC rollouts. However, they lacked stratification for stroke risk as well as data on clinical and economic outcomes, and they had relatively small sample sizes [34–38]. Moreover, they differed in patient populations and results. For example, a national registry study and a Medicare dataset study (2013–2016 and 2010–2017, respectively) reported increasing trends in OAC use during data years after guideline implementation [36, 37]. In contrast, a VA dataset study found no appreciable change in OAC use (2007–2016) [38]. Thus, more comprehensive, chronological evidence is needed to characterize associations between guideline introduction, DOAC uptake, and patient outcomes among US patients with NVAF and high stroke risk.

Therefore, the authors undertook this retrospective analysis of temporal trends in OAC use, stroke/SE and MB incidence, and healthcare costs among Medicare patients with NVAF and high stroke risk scores from 2013–2016.

## Methods

### Data source

This retrospective cohort study utilized the 100% US Centers for Medicare & Medicaid Services (CMS) fee-for-service Medicare dataset from January 1, 2012 through December 31, 2017. Data included medical and pharmacy claims from Medicare Parts A [hospitalization, skilled nursing facility (SNF), and hospice care], B [outpatient care, durable medical equipment (DME), home health agency (HHA)], and D (prescription drug coverage). Medical claims were evaluated using the International Classification of Diseases, Ninth and Tenth Revision, Clinical Modification (ICD-9-CM/ICD-10-CM/ICD-10-PCS) diagnosis and procedure codes as well as Health Care Common Procedure Coding System (HCPCS) and Current Procedural Terminology codes; pharmacy claims were identified using NDC codes.

### Patient selection

For each year of the identification period (2013–2016), patients aged ≥ 65 years with ≥ 1 inpatient or ≥ 2 outpatient claims (≥ 7 days apart and within 365 days) for AF (ICD-9 427.3; ICD-10 I48 any diagnosis position) were selected within calendar year cohorts. The first observed AF diagnosis of that year was designated as the index date. For each calendar year cohort, patients were required to have continuous medical and pharmacy coverage for 12 months prior to the index date through 12 months after the index date (patients who died during follow-up were included to avoid selection bias). Patients were required to have a CHA_2_DS_2_ -VASc score ≥ 2 and were excluded if they had rheumatic mitral valvular heart disease or valve replacement procedure at any time before or on the day of AF diagnosis (Supplemental Table 1).

### Baseline characteristics

Age, sex, and race were ascertained on the index date (AF diagnosis), and baseline clinical characteristics were examined during the 12-month pre-index period within each calendar year cohort. Mean CHA_2_DS_2_-VASc and modified HAS-BLED scores were calculated to evaluate risk of stroke and major bleeding, respectively [39]. The CHA_2_DS_2_-VASc score evaluates stroke risk on a 0–9 scale calculated based on 8 weighted factors: congestive heart failure; hypertension; age ≥ 75 years; diabetes mellitus; history of stroke, thromboembolism, or transient ischemic attack; vascular disease; age 65–74 years; and sex category [39]. The modified HAS-BLED score estimates the 1-year risk of MB on a 0–8 scale calculated based on 6 weighted factors: hypertension, abnormal kidney and/or liver function, stroke, bleeding, age > 65 years, and prior use of alcohol/drugs or medications associated with bleeding risk (the labile international normalized ratio, lab values, and self-reported alcohol consumption levels included in the standard HAS-BLED score were not available) [40].

### Outcomes

Outcomes were examined during the follow-up period, which was defined as the index date through 12 subsequent months for each calendar-year cohort, with all observed outcomes recorded for that cohort. Reported treatment outcomes included the overall proportions of patients prescribed OACs on or after the index date, stratified by OAC type (warfarin, dabigatran, rivaroxaban, apixaban, and edoxaban) and the proportion of patients not prescribed OACs during the follow-up period.

Clinical and economic outcomes were examined during the 12-month follow-up period from NVAF diagnosis (index date) until death or end of follow-up. The clinical outcomes were incidence rates of stroke/SE and MB. These outcomes were defined by hospitalizations with a principal diagnosis for stroke/SE or MB, respectively. Stroke/SE included ischemic stroke, hemorrhagic stroke, and systemic embolism. MB included gastrointestinal bleeding, intracranial hemorrhage, and bleeding at other key sites [41, 42]. Economic outcomes included all-cause total healthcare costs, which were comprised of inpatient, outpatient / emergency room (ER), pharmacy, and other costs (DME, HHA, hospice, SNF) from medical/pharmacy claims.

### Statistical analysis

All study variables were described with standard summary statistics among calendar year cohorts: numbers and percentages were reported for categorical variables; means and standard deviations (SDs) were reported for continuous variables. Clinical outcome incidence rates were presented per 100 person-years, calculated as the number of patients with events of interest divided by time at risk for developing the event (divided by 100) within the year of interest.

The mean healthcare costs were reported per person per month (PPPM) and calculated by adding the costs from inpatient, outpatient/ER, pharmacy, and other (DME, SNF, hospice, and HHA) settings. The costs were adjusted to 2017 US dollars using the medical care component of the Consumer Price Index from the US Department of Labor. Data analysis was performed using statistical software SAS version 9.4 (SAS Institute Inc., Cary, NC, USA).

### Institutional review board approval

As this study did not involve the collection, use, or transmittal of individually identifiable data, it was exempt from Institutional Review Board review. Both the datasets and the security of the offices where analysis was completed (and where the datasets are kept) meet the requirements of the Health Insurance Portability and Accountability Act of 1996.

## Results

### Study population

After selection criteria application, there was a progressive increase in the number of patients with NVAF and a CHA_2_DS_2_-VASc score of ≥ 2 each calendar year from approximately 1.6 million in 2013 to approximately 1.9 million in 2016 (Supplemental Table 2). Patient demographics were generally consistent across the four calendar year cohorts; mean ages were ~ 80 years, and most patients were white (~ 90%). The proportion of female patients decreased slightly over the study period (57–54%). Across the calendar years, the mean CHA_2_DS_2_-VASc score was approximately 4.6 and the mean modified HAS-BLED score was 3.2 (Table 1).

**Table 1:** Baseline patient characteristics of Medicare patients with NVAF at increased stroke risk (2013–2016)

	2013		2014		2015		2016	
	N/Mean	%/SD	N/Mean	%/SD	N/Mean	%/SD	N/Mean	%/SD
Sample size	1,611,036		1,747,852		1,830,882		1,881,432	
Age	80.5	8.0	80.1	7.9	80.0	7.9	79.9	7.9
65–74	423,819	26.3%	483,953	27.7%	518,655	28.3%	544,968	29.0%
75–79	321,506	20.0%	361,368	20.7%	383,248	20.9%	401,224	21.3%
≥ 80	865,711	53.7%	902,531	51.6%	928,979	50.7%	935,240	49.7%
Gender								
Male	696,886	43.3%	786,463	45.0%	840,011	45.9%	874,667	46.5%
Female	914,150	56.7%	961,389	55.0%	990,871	54.1%	1,006,765	53.5%
Race								
White	1,443,459	89.6%	1,571,060	89.9%	1,646,767	89.9%	1,692,247	89.9%
African American	92,465	5.7%	98,761	5.7%	101,225	5.5%	102,014	5.4%
Other	75,112	4.7%	78,031	4.5%	82,890	4.5%	87,171	4.6%
Baseline comorbidity^a^								
History of bleed	372,329	23.1%	391,467	22.4%	404,298	22.1%	404,371	21.5%
History of stroke/SE	187,655	11.6%	187,656	10.7%	193,175	10.6%	194,008	10.3%
Charlson comorbidity index	3.1	2.8	3.0	2.7	3.0	2.7	3.1	2.8
CHA_2_DS_2_ –VASc score	4.7	1.7	4.6	1.7	4.6	1.7	4.5	1.6
HAS-BLED score^b^	3.2	1.2	3.1	1.2	3.2	1.2	3.2	1.3

CHA_2_DS_2_-VASc: congestive heart failure, hypertension, aged ≥ 75 years, diabetes mellitus, prior stroke or transient ischemic attack or thromboembolism, vascular disease, aged 65–74 years, sex category

*DOAC* direct oral anticoagulant, *HAS-BLED* hypertension, abnormal renal or liver function, stroke, bleeding, labile international normalized ratios, elderly, drugs or alcohol, *NVAF* non-valvular atrial fibrillation, *SD* standard deviation, *SE* systemic embolism

^a^Comorbidities were defined using diagnosis codes in any claim position

^b^Modified HAS-BLED scores did not include labile international normalized ratios, certain lab values, or patient-reported alcohol consumption levels.

### Proportions of oral anticoagulant treatment

**Fig. 1:**
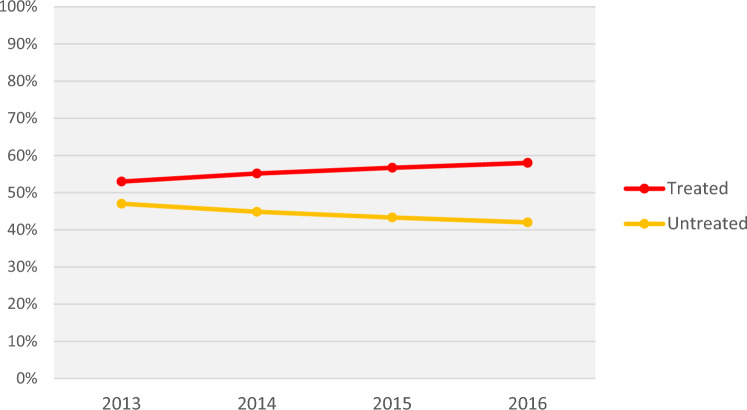
Total OAC treatment proportions among Medicare patients with NVAF at increased stroke risk from 2013–2016. For each calendar year, OAC prescriptions were captured on the NVAF diagnosis date or during the 12 months after the NVAF diagnosis. *OAC* oral anticoagulant, *NVAF* non-valvular atrial fibrillation

**Fig. 2:**
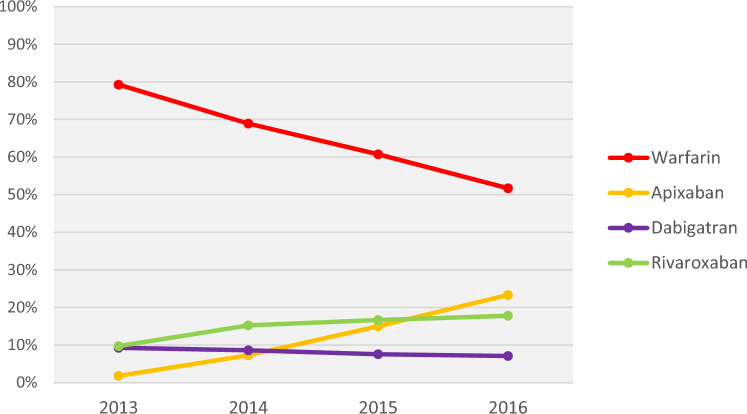
Type of OAC treatment among OAC prescribed medicare patients with NVAF at increased stroke risk from 2013–2016. For each calendar year, OAC prescriptions were captured on the NVAF diagnosis date or during the 12 months after the NVAF diagnosis. *OAC* oral anticoagulant, *NVAF* non-valvular atrial fibrillation

Over the 4 years from 2013 to 2016, the proportion of eligible patients not treated with OACs slightly but progressively decreased from 47 to 42% but till remained high (the proportion of overall OAC-treated patients increased from 53 to 58%; Fig. 1). Among the OAC-treated from 2013–2016 (Fig. 2), DOAC prescriptions also became progressively more common, with proportions of patients prescribed warfarin progressively decreasing from 79 to 52%; DOACs progressively increasing from 21 to 48%. Among DOACs, proportions for apixaban and rivaroxaban progressively increased (from 2 to 23% and 10% to 18%, respectively) while dabigatran progressively decreased from 9 to 7%. Edoxaban prescriptions began in 2015 and remained below 1% through 2016 (data not shown).

### Incidence of stroke/SE and MB

**Fig. 3:**
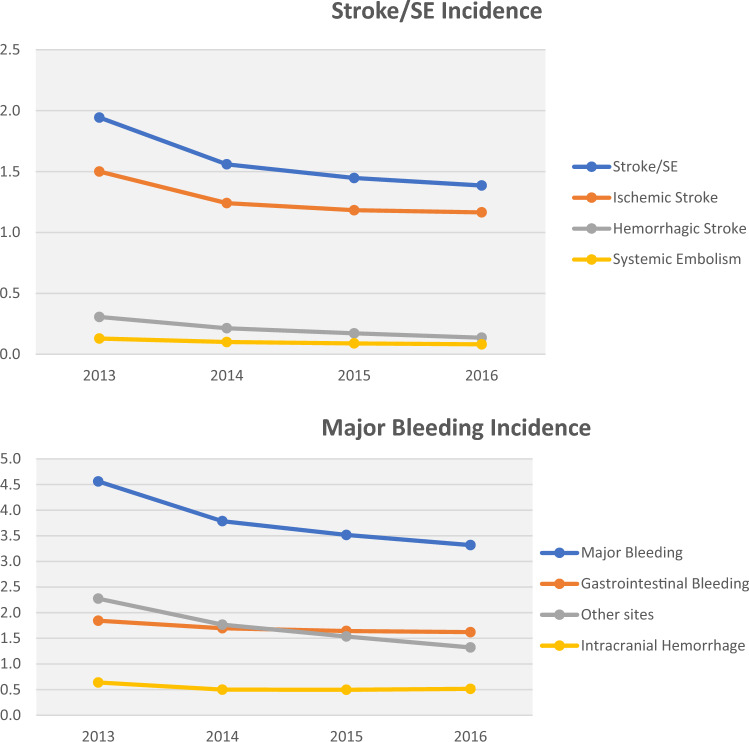
Incidence rates of stroke/SE and MB per 100 person years among Medicare patients with NVAF at increased stroke risk from 2013–2016. Incidence rates of stroke/systemic embolism and major bleeding were calculated per 100 person-years. For each calendar year, stroke/systemic embolism and major bleeding were measured on the non-valvular atrial fibrillation diagnosis date or during the 12 months after the non-valvular atrial fibrillation diagnosis, regardless of oral anticoagulant treatment

The observed temporal trends in treatment patterns coincided with a generally progressive decrease in the yearly incidence rates of stroke/SE and MB from 2013–2016. Incidence rates of stroke/SE decreased from 1.9 to 1.4 per 100 person years (Fig. 3). All types of stroke/SE decreased, including hemorrhagic stroke (0.3–0.1 per 100 person years), ischemic stroke (1.5–1.2 per 100 person years), and SE (0.13–0.08 per 100 person years). A similar trend was observed for MB, which decreased from 4.6 to 3.3 per 100 person years. A reduction was observed for all types of MB, including ICH, which decreased from 0.6 to 0.5 per 100 person years (Fig. 3).

### Healthcare costs

**Fig. 4:**
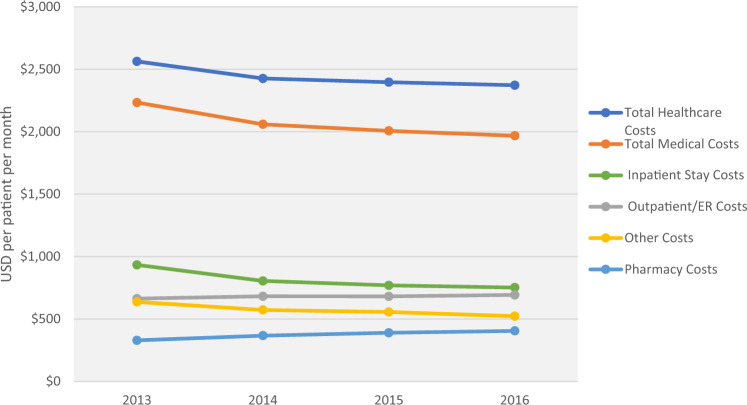
Follow-up total healthcare costs (PPPM) among Medicare patients with NVAF at increased stroke risk from 2013–2016. Total costs were calculated per patient per month during the 12 months on or after NVAF diagnosis, regardless of OAC treatment. Total costs (adjusted to 2017 dollars) included medical and pharmacy costs, of which medical costs include inpatient, outpatient/ER, and other costs (durable medical equipment, home health agency, hospice, and skilled nursing facility). *NVAF* non-valvular atrial fibrillation, *PPPM* per patient per month, *USD* United States Dollars

Although there was some directional variation, overall follow-up total all-cause healthcare costs (outpatient/ER, inpatient, other, pharmacy) also progressively decreased from $2563 PPPM in 2013 to $2372 PPPM in 2016. Inpatient costs progressively decreased from $933 to $752 PPPM, while outpatient/ER costs remained relatively consistent across the 4 years ($664–$693); pharmacy costs increased from $329 to $405 PPPM (Fig. 4).

## Discussion

This study provides a comprehensive, temporal evaluation of OAC treatment and outcome trends in the US after the 2014 introduction of clinical guideline recommendations for the use of the CHA_2_DS_2_-VASc risk assessment tool and preference of DOACs over warfarin for patients with NVAF at increased stroke risk. The findings describe a marked uptake of DOACs (from roughly one-fifth to half of OAC-treated patients) in place of warfarin. This shift coincided with generally progressive reductions in stroke/SE and MB incidence (including a steep reduction when guidelines first were implemented) as well as healthcare costs during the 4-year study period. However, we observed a smaller increase in overall OAC utilization (53–58%) within the context of persistently high proportions of eligible patients not prescribed OACs (47–42%).

Our findings add to the timeline of real-world observations of OAC treatment for NVAF after the introduction of DOACs and are generally consistent in directionality [19–23, 25–31]. In their cross-sectional registry study of > 400,000 patients with AF from 2008–2012, Hsu et al. found that ~ 45% were treated with an OAC over the entire study period—among whom 90% were prescribed warfarin, 8% dabigatran (2010 FDA approval), and 2% rivaroxaban (2011 FDA approval) [20]. Apixaban (2012 FDA approval) was not included. As compared with our results for 2013 (OAC: 53%; warfarin: 79%; dabigatran: 9%, rivaroxaban: 10%, apixaban: 2%), and given that the study population in Hsu et al. was not stratified by calendar year and was somewhat younger with lower stroke risk [mean age: 71 years (vs 80.5); CHA_2_DS_2_–VASc score: 3.7 (vs 4.7)], the two sets of results generally align to describe an overall progression of both increased DOAC use and increased overall OAC use. Similar overlapping temporal trends were also observed in the US by Ashburner et al. (2010–2015), Alcusky et al. (2011–2016), Steinberg et al. (2013–2016), and Zhu et al. (2010–2017) [34–37]. While Rose et al. (2007–2016) also observed a marked uptake of DOAC use during a similar period, they did not observe substantive changes in overall OAC use. However, this discrepancy may be attributable to differing patient characteristics of the VA database [38]. Upward trends have also been observed in Europe, Australia, and East Asia [25–33]. Thus, while continued research across varying patient populations is necessary, the preponderance of existing evidence is consistent.

Notably, the overall OAC use we observed is generally consistent with US real-world studies that suggest possible underutilization at earlier periods, during a generally similar period (2010–2015), and more recently (2018) [18–22] [43], More recent data in particular raise questions about why underuse persists despite guideline implementation. Ko et al. found that among Medicare beneficiaries, 67.1% of patients with incident AF in 2020 had not initiated an OAC within 12 months of diagnosis [44]. While legitimate considerations for contraindications due to comorbidity, polypharmacy, extremely high bleeding risk, or unobserved antiplatelet or aspirin prescription may play a role, there may also be some clinical inertia attributable to inappropriate concerns regarding older age, sex, frailty, or moderate bleeding risk, as well as suboptimal guideline awareness [23, 24, 45]. A study by Navar et al. suggests differences at the provider‐ and health-system‐level are more of a driving factor in the underutilization of DOAC treatment than patient level factors among patients with NVAF [45]. Regardless, more research is necessary to help guide clinical decision-making.

There are scarce data currently available that examine temporal trends in stroke/SE and MB among patients with NVAF during comparable periods in the US. Hohnloser et al. conducted a German retrospective claims study that found a progressive 24% reduction in adjusted stroke incidence from 2011–2016, in parallel with DOAC uptake following the implementation of similar clinical guidelines in 2013 [27]. Although populations and payer systems differed, Hohnloser et al. found a decrease in crude stroke/SE rates (1.9–1.6 per 100 person years) roughly comparable to our (unadjusted) results (1.9–1.4 per 100 person years). However, crude MB rates in Hohnloser et al. increased slightly over the period, from 1.9 to 2.1 per 100 person years, in contrast to 4.6–3.3 per 100 person years observed in our study. Moreover, while studies by Maggioni et al. and Narita et al. found reduced proportions of hospitalizations for stroke in parallel with DOAC uptake in Italy and Japan, respectively [30, 33], Maggioni et al. found a slight increase in hospitalizations for MB. Ding et al. observed the stroke trend in Sweden from 2001–2020 noting similar results in stroke reduction and increase in DOAC use, but highlighted that as of 2020 one in four strokes among elderly Swedish patients was preceded by or concurrent with an AF diagnosis [46]. While these discrepancies may be attributable to differing patient characteristics and specifics of national-level guidelines, they warrant more research to verify our findings in US datasets stratified by year.

To the best of our knowledge, there are no previous data on US costs for patients with NVAF at the national level stratified by year. As a general benchmark, an internal Medicare retrospective study of all Medicare patients with AF from 2004–2008 found approximate costs of $2,000 PPPM (based on yearly costs of $24,000 in 2009 US dollars) [47]. A recent study found that patients with AF incurred ~ $25,000 PPPY higher healthcare costs compared to patients without AF [48]. Another retrospective claims analysis of Medicare patients treated with OACs between 2013–2014 found a range of $3180 to $3878 PPPM (2014 US dollars) [49] as compared with $2563 PPPM among all patients with NVAF in our 2013 cohort (2017 US dollars), reflecting higher costs among the treated. Notably, this study was among several retrospective analyses of Medicare patients that showed lower overall healthcare costs among patients prescribed DOACs as compared with warfarin. Given the increase in proportions of those prescribed DOACs over our study period, this may have contributed to the observed reduction in costs. The observed reduction in cost is driven by the reduced inpatient cost over time, which is likely the result of reduced stroke and/or bleeding events due to patients switching to DOACs over our study period. Despite the coinciding trends of increased DOAC use, reduced events and reduced inpatient cost over time, further research is needed to determine a causal relationship between increased DOAC use and reduced events and reduced cost. Results warrant continued research that adjusts for patient characteristics between calendar year cohorts.

Taken together and in context with similar trends observed abroad, these results provide insight on the interplay between guideline introduction and uptake time for new agents that can help inform decision-making for clinicians, payers, and policy makers. Future research should investigate the reasons for clinical practices apparently contrary to the evidence and guidelines.

## Strengths and limitations

This is the first study of its kind stratified by year to analyze a large, highly representative dataset at the national level; the results provide a highly inclusive and comprehensive descriptive observation of the treatment landscape during a time of evolution of oral anticoagulation in the US. Nonetheless, results should be interpreted in the context of certain limitations. For instance, all claims data are collected for administrative purposes rather than research and therefore lack certain clinical information such as actual medication administration as well as observation of samples and over-the-counter medication. Moreover, claims are subject to coding discrepancies. As with all retrospective analyses, interpretation of these data is limited to observation of associations rather than inference of causality, and observed coinciding trends in this study should be interpreted accordingly. Other limitations specific to this study include the absence of information on treatment switching and discontinuation, as patients were assigned to OAC-treated cohorts based only on the first prescription received during the follow-up period. In addition, this study was performed in a Medicare population aged ≥ 65 years; results may not be generalizable to younger patients, those at lesser risk, and those with other forms of insurance. Also notable, the clinical guidelines for OAC treatment among patients with NVAF were not yet implemented during the first calendar year examined and have changed again since the end of the study period. We reported treatment among both male and female patients with NVAF and a CHA_2_DS_2_-VASc score of ≥ 2 in alignment with 2014 guidelines; however, the most recent guidelines recommend treatment for males with a CHA_2_DS_2_-VASc score of ≥ 2 and females with a CHA_2_DS_2_-VASc score of ≥ 3.^15^ Finally, the calendar year cohorts were not balanced for variation in patient characteristics, so results should be interpreted accordingly. Nonetheless, the consistency of observed progressive directional trends with similar findings in other countries warrant continued research.

## Conclusion

This study evaluated temporal trends between 2013 and 2016 in OAC treatment and related clinical and economic outcomes among a large Medicare patient population with NVAF at increased risk of stroke. The results reveal rapid and progressive temporal trends toward DOAC uptake supplanting warfarin prescriptions, which chronologically follow clinical guidelines (2014) calling for the use of DOAC prescriptions instead of warfarin for patients with NVAF at high risk of stroke. This trend coincided with progressive reductions in stroke/SE, MB, and healthcare costs. However, the proportions of overall OAC use increased by less than 10%, possibly indicating persistent OAC underutilization. Further study is needed to determine where gaps in treatment may remain.

### Supplementary Information

Below is the link to the electronic supplementary material.

Supplementary file1 (DOCX 20 KB)

## Data Availability

The raw insurance claims data used for this study originate from Medicare data, which are available from the Centers for Medicare and Medicaid through ResDAC (https://www.resdac.org/). Other researchers could access the data through ResDAC, and the inclusion criteria specified in the Methods section would allow them to identify the same cohort of patients we used for these analyses.
