# Supplementary material for: Temporal trends in anticoagulation use and clinical outcomes among medicare beneficiaries with non-valvular atrial fibrillation
Source: J Thromb Thrombolysis. 2023 Aug 2;57(1):1–10. doi: 10.1007/s11239-023-02838-2 (PMC10830709; doi:10.1007/s11239-023-02838-2)
Supplement: Supplementary file 1 — Supplementary file1 (DOCX 20 KB) [file 11239_2023_2838_MOESM1_ESM.docx]

**Supplemental Material**

**Supplemental Table 1. Diagnosis and Procedure Codes for Selection Criteria**

| **Diagnosis** | **ICD-9-CM/PCS Codes** | **ICD-10-CM/PCS Codes** |
| --- | --- | --- |
| Atrial Fibrillation or Flutter | 427.31, 427.32 | I480, I481, I482, I483, I484, I4891, I4892 |
| Rheumatic Mitral Valvular Heart Disease | 394.0, 394.1, 394.2, 394.9, 396.0, 396.1, 396.8, 396.9, 424.0, 745.xx | I05.0, I05.1, I05.2, I05.8, I05.9, I08.0, I08.8, I08.9, I34.0, I34.1, I34.2, I34.8, I34.9, Q21.3, Z95.2, Z95.3, Z95.4 |
| Valve Replacement Procedure | 35.05-35.09, 35.20-35.28, 35.97 | 02RF07Z, 02RF08Z, 02RF0JZ, 02RF0KZ, 02RF37H, 02RF37Z, 02RF38H, 02RF38Z, 02RF3JH, 02RF3JZ, 02RF3KH, 02RF3KZ, 02RF47Z, 02RF48Z, 02RF4JZ, 02RF4KZ, 02RG07Z, 02RG08Z, 02RG0JZ, 02RG0KZ, 02RG37H, 02RG37Z, 02RG38H, 02RG38Z, 02RG3JH, 02RG3JZ, 02RG3KH, 02RG3KZ, 02RG47Z, 02RG48Z, 02RG4JZ, 02RG4KZ, 02RH07Z, 02RH08Z, 02RH0JZ, 02RH0KZ, 02RH37H, 02RH37Z, 02RH38H, 02RH38Z, 02RH3JH, 02RH3JZ, 02RH3KH, 02RH3KZ, 02RH47Z, 02RH48Z, 02RH4JZ, 02RH4KZ, 02RJ07Z, 02RJ08Z, 02RJ0JZ, 02RJ0KZ, 02RJ47Z, 02RJ48Z, 02RJ4JZ, 02RJ4KZ, 02UG3JZ, X2RF032, X2RF332, X2RF432 |

**Supplemental Table 2. NVAF patient selection for 2013-2016**

| **Selection Criteria** | **2013** | **2014** | **2015** | **2016** |
| --- | --- | --- | --- | --- |
|  | **Sample Size** | **Sample Size** | **Sample Size** | **Sample Size** |
| a) Patients had at least 1 inpatient or 2 outpatient claims at least 7 days apart and within 365 days for AF (atrial fibrillation; any diagnosis) | 4,380,075 | 4,455,589 | 4,578,091 | 4,701,639 |
| b) Patients had continuous Medicare enrollment with medical and pharmacy benefits for 12 months prior to the index date (baseline period) until 12 months after the index date (including the index date; follow-up period) | 2,361,907 | 2,722,045 | 2,892,168 | 3,053,671 |
| c) Patients were aged ≥65 years on the index date | 2,198,892 | 2,547,471 | 2,711,113 | 2,868,871 |
| d) Patients had no evidence of rheumatic mitral valvular heart disease or valve replacement procedure any time before or on the index date. | 1,641,855 | 1,784,282 | 1,871,673 | 1,926,335 |
| e) Patients had a CHA_2_DS_2_-VASc ≥2 during the baseline period | 1,611,036 | 1,747,852 | 1,830,882 | 1,881,432 |
| **Final Sample Size** | 1,611,036 | 1,747,852 | 1,830,882 | 1,881,432 |
